# Cerebrovascular risk factors associated with ischemic stroke in a young non-diabetic and non-hypertensive population: a retrospective case-control study

**DOI:** 10.1186/s12883-020-02005-7

**Published:** 2020-11-23

**Authors:** Nan Zhang, Lin Zhang, Qiu Wang, Jingwei Zhao, Jia Liu, Guang Wang

**Affiliations:** Department of Endocrinology, Beijing Chao-yang Hospital, Capital Medical University, Beijing, 100020 China

**Keywords:** Ischemic stroke, Risk factors, Young adults, Early-onset, Young-onset

## Abstract

**Background:**

Globally, rates of ischemic stroke (IS) have been rising among young adults. This study was designed to identify risk factors associated with IS incidence in young adults unaffected by hypertension or diabetes.

**Methods:**

This was a retrospective case-control study of early-onset IS patients without diabetes and hypertension. Control patients were matched with healthy individuals based upon sex, age (±2 years), and BMI (±3 kg/m^2^) at a 1:3 ratio. Sociodemographic, clinical, and risk factor-related data pertaining to these patients was collected. The association between these risk factors and IS incidence was then assessed using conditional logistic regression models.

**Results:**

We recruited 60 IS patients and 180 controls with mean ages of 44.37 ± 4.68 and 44.31 ± 4.71 years, respectively, for this study. Relative to controls, IS patients had significantly higher total cholesterol (TG), homocysteine (HCY), white blood cell (WBC), absolute neutrophil count (ANC), systolic blood pressure (SBP), and diastolic blood pressure (DBP) levels, and significantly lower high-density lipoprotein cholesterol (HDL-C) and triglyceride cholesterol (TC), free triiodothyronine (FT3), and free thyroxine (FT4) levels (all *P* < 0.05). After controlling for potential confounding factors, HCY and ANC were found to be significantly positively associated with IS incidence (OR 1.518, 95%CI 1.165–1.977, *P* = 0.002 and OR 2.418, 95%CI 1.061–5.511, *P* = 0.036, respectively), whereas HDL-C and FT3 levels were negatively correlated with IS incidence (OR 0.001, 95%CI 0.000–0.083, *P* = 0.003 and OR 0.053, 95%CI 0.008–0.326, *P* = 0.002, respectively).

**Conclusions:**

In young non-diabetic and non-hypertensive patients, lower HDL-C and FT3 levels and higher HCY and ANC levels may be associated with an elevated risk of IS. Additional prospective studies of large patient cohorts will be essential to validate these findings.

## Background

Ischemic stroke (IS) is a serious condition associated with high morbidity and mortality rates throughout the world. Between 10 and 18% of IS episodes first occur in persons under the age of 50 [1, 2], and such incidence is termed early/young-onset stroke or stroke in young adults [3–5]. IS incidence in younger adults can incur high healthcare costs and can significantly decrease labor productivity [6]. As rates of IS among young adults are rising faster than rates among older adults, the global burden associated with this condition is likely to continue rising in the immediate future [7, 8].

Traditionally, risk factors associated with over 80% of IS incidence among young adults include smoking, hypertension, obesity, diabetes, and dyslipidemia,^9^ with approximately 10 and 35% of these patients being affected by diabetes and hypertension, respectively [9, 10]. Diabetes and its cardiovascular events have a clear inflammatory pathogenesis involved with an adipose-inflammatory vascular axis predisposing to atherosclerosis [11], oxidative stress [12] and atrial fibrillation due to cardiac nervous autonomic dysfunction [13]. Moreover, hypertension can increase the risk of these diabetic vascular complications [14]. Even patients unaffected by these conditions still suffer from IS in some cases, however, with roughly 33% of IS cases in this age group being cryptogenous [15]. Identifying risk factors associated with IS in these patients may facilitate better preventative or interventional treatment. As such, the present study was designed to identify risk factors associated with IS incidence in young adults unaffected by hypertension and diabetes.

## Methods

### Study design and participants

Between March and October 2019, 60 non-diabetic and non-hypertensive inpatients at Beijing Chao-yang Hospital that had suffered their first-ever incidence of acute IS were recruited for this study (age: 32–50 years). IS as defined as sudden onset neurological dysfunction associated with an infarcted site that was detected via brain magnetic resonance imaging (MRI). A healthy control population that was matched according to age (±2-years), sex, and BMI (±3.0 kg/m^2^) were recruited randomly from among patients undergoing physical examinations at our hospital. Recruitment was conducted at a 1:3 case to control ratio. Patients were excluded from this study if they suffered from known hypertension, diabetes, cancer, autoimmunity, thyroid disease, encephalitis, head trauma, cerebral hemorrhage, severe multiple organ dysfunction, infections, severe liver or kidney diseases, or pregnancy. The Medical Ethics Committee of Beijing Chaoyang Hospital approved this study, which was consistent with the Declaration of Helsinki, and the requirement for written informed consent was waived due to the retrospective design. Patient data have been de-identified upon data collection.

### Measurements

For all patients, the following data were recorded: sex, age, BMI, smoking status, SBP, DBP, fasting blood glucose (FBG), creatinine (CR), uric acid (UA), HCY, TC, TG, HDL-C, low-density lipoprotein cholesterol (LDL-C), FT3, FT4, and thyroid-stimulating hormone (TSH) levels, as well as WBC, ANC, absolute lymphocyte count (ALC), platelet (PLT), and hemoglobin (HGB) values. All IS patients underwent routine testing include brain MRI scans, chest X-ray, and cardiac tests including electrocardiography and transthoracic echocardiograms in an effort to detect any potential atrial fibrillation, ischemic heart disease, or rheumatic heart disease. Other testing was conducted as appropriate in order to ensure that patients met with study inclusion/exclusion criteria.

Following overnight fasting, blood samples were collected from the antecubital vein of each patient and were analyzed with an Abbott Architect i2000 (Abbott Diagnostics, IL, USA). The same medical staff measured the weight and height of all patients, and BMI (in kg/m^2^) was calculated based on these values. The same 3.0 T Siemens scanner (Erlangen, Germany) was used for all MRI scans.

### Statistical analysis

SPSS 24.0 (SPSS, IL, USA) was used for all statistical testing. The Kolmogorov-Smirnov test was used to assess the normality of distributed data, with normally and non-normally distributed data being expressed as means ± standard deviation (SD) and medians with interquartile ranges (IQR). Data were compared between case and control groups using chi-squared tests, Student’s t-tests, or Mann-Whitney U tests as appropriate, and univariate odds ratios (ORs) for these risk factors were calculated. Those risk factors that were significantly associated with IS incidence in univariate analyses were then incorporated into a multivariate conditional logistic regression model, after which adjusted ORs and 95% confidence intervals were estimated. For this model, a forward stepwise logistic regression approach with a *P* < 0.10 variable removal level was used. A two-sided *P* < 0.05 was the significance threshold for this study.

## Results

### Patient baseline characteristics

Baseline data pertaining to the control and IS patient groups in this study are compiled in Table 1. In total, 51/60 IS patients were male, a history of cigarette smoking was reported in 68.3% of these patients, and the mean age at time of stroke was 44.31 ± 4.71 years. There were no significant differences in age, sex, or BMI between these two patient groups, nor were any differences detected in serum TSH, LDL-C, FBG, CR, UA, ALC, HGB, or PLT when comparing these groups (all *P* > 0.05). However, IS patients exhibited significantly lower HDL-C and TC levels relative to controls [0.95 (0.62–1.59) vs. 1.16 (0.69–2.29) mmol/L, 4.39 ± 1.08 vs. 4.97 ± 0.78 mmol/L, respectively, *P* < 0.001]. Levels of serum FT3 and FT4 were also significantly lower in IS patients relative to controls, while TG, HCY, WBC, ANC, SBP, and DBP values were all significantly elevated among IS patients (all *P* < 0 .05).

**Table 1:** The baseline characteristics of the control and IS groups

Parameters	Control group (*n* = 180)	IS group (*n* = 60)	*P*
Age, y	44.31 ± 4.71	44.37 ± 4.68	.918
BMI, kg/m^2^	25.65 ± 2.69	25.86 ± 2.57	.593
Gender, Females/Males	27/153	9/51	1.000
Smoker/non-smoker	0	41/19	.000
FT3, pmol/L	5.42 ± 0.54	4.72 ± 0.69	.000
FT4, pmol/L	16.38 ± 2.18	12.26 ± 2.91	.000
TSH, mIU/L	1.79 (0.59–5.38)	1.58 (0.23–6.5)	.548
HDL-C, mmol/L	1.16 (0.69–2.29)	0.95 (0.62–1.59)	.000
LDL-C, mmol/L	2.80 ± 0.65	2.72 ± 0.91	.117
TC, mmol/L	4.97 ± 0.78	4.39 ± 1.08	.000
TG, mmol/L	1.41 (0.43–3.85)	1.58 (0.61–6.25)	.032
FBG, mmol/L	5.49 ± 0.50	5.48 ± 1.06	.080
CR, μmol/L	69.79 ± 11.82	68.45 ± 11.68	.289
UA, μmol/L	337.33 (145.00–473.95)	325.50 (191.00–542.00)	.445
HCY, μmol/L	10.42 (6.25–17.83)	16.08 (5.00–136.87)	.000
WBC, 10^9^/L	6.04 (3.28–10.09)	7.03 (4.50–19.6)	.001
ANC, 10^9^/L	3.40 (1.18–6.60)	4.18 (2.26–17.70)	.000
ALC, 10^9^/L	2.05 (0.78–4.76)	2.08 (1.02–4.90)	.949
HGB, g/L	151.12 ± 14.97	148.17 ± 16.81	.097
PLT, 10^9^/L	217.23 ± 42.66	231.43 ± 56.99	.109
SBP, mmHg	123.41 ± 13.07	137.63 ± 17.89	.000
DBP, mmHg	77.20 ± 10.73	85.87 ± 12.81	.000

Data are means ± S.D. unless indicated otherwise. TSH, HDL-C, TG, UA, HCY, WBC, ANC and ALC are shown as median, upper and lower quartiles. *P*: the stroke group vs the control group, analyzed by chi-square test, independent Student’s t-test or the Mann–Whitney test using SPSS 24.0. IS, ischemic stroke; *BMI* body mass index; *FT3* free triiodothyronine; *FT4* free thyroxine; *TSH* thyroid-stimulating hormone; *HDL-C* high-density lipoprotein cholesterol; *LDL-C* low-density lipoprotein cholesterol; *TC* triglyceride cholesterol; *TG* total cholesterol; *FBG* fasting blood glucose; *CR* creatinine; *UA* uric acid; *HCY* Homocysteine; *WBC* white blood cell; *ANC* absolute neutrophil count; *ALC* absolute lymphocyte count; *HGB* hemoglobin, *PLT* platelet; *SBP* systolic blood pressure; *DBP* diastolic blood pressure

### Identification of risk factors associated with early-onset IS

Univariate logistic regression analyses were initially used to identify risk factors associated with IS incidence (Table 2), revealing FT3, FT4, HDL-C, TC, TG, HCY, WBC, ANC, PLT, SBP, and DBP values to all be significantly associated with such incidence. Subsequent multivariate conditional logistic regression analysis revealed that HDL-C, FT3, HCY, and ANC levels were all independently associated with IS risk (Table 3). Both HCY and ANC levels were positively correlated with IS incidence with respective ORs of 1.518 (95%CI 1.165–1.977, *P* = 0.002) and 2.418 (95%CI 1.061–5.511, *P* = 0.036). In contrast, HDL-C and FT3 levels were significantly negatively correlated with IS rates (OR 0.001, 95%CI 0.000–0.083, *P* = 0.003 and OR 0.053, 95%CI 0.008–0.326, *P* = 0.002, respectively).

**Table 2:** Univariate logistic regression model of risk factors for IS in non-diabetic and non-hypertensive young population

Parameters	B	SE	Wald	*P*	Exp (B)	95%CI
Age, y	.003	.032	.006	.937	1.003	.942–1.067
Gender	.000	.439	.000	1.000	1.000	.423–2.362
BMI, kg/m^2^	.030	.056	.288	.592	1.031	.923–1.151
Smoking	−23.446	6277.089	.000	.997	.000	.000-
FT3, pmol/L	−1.954	.311	39.493	.000	.142	.077–.261
FT4, pmol/L	−.665	.090	54.669	.000	.514	.431–.613
TSH, mIU/L	.192	.144	1.792	.181	1.212	.915–1.606
HDL-C, mmol/L	−4.434	.832	28.376	.000	.012	.002–.061
LDL-C, mmol/L	−.166	.213	.607	.436	.847	.559–1.285
TC, mmol/L	−.804	.194	17.114	.000	.447	.306–.655
TG, mmol/L	.449	.175	6.592	.010	1.566	1.112–2.206
FBG, mmol/L	−.035	.219	.026	.872	.965	.628–1.484
CR, μmol/L	−.010	.013	.580	.446	.990	.966–1.015
UA, μmol/L	.000	.002	.016	.900	1.000	.996–1.005
HCY, μmol/L	.352	.063	31.443	.000	1.422	1.257–1.608
WBC, 10^9^/L	.351	.101	12.132	.000	1.420	1.166–1.730
ANC, 10^9^/L	.506	.136	13.861	.000	1.659	1.271–2.167
ALC, 10^9^/L	.096	.237	.165	.685	1.101	.691–1.754
HGB, g/L	−.012	.009	1.628	.202	.988	.970–1.006
PLT, 10^9^/L	.006	.003	4.005	.045	1.006	1.000–1.013
SBP, mmHg	.066	.012	28.752	.000	1.068	1.043–1.094
DBP, mmHg	.064	.014	21.086	.000	1.066	1.037–1.096

*B* regression coefficient; *SE* standard error; Exp (B) indicates odds ratio (OR); *CI* indicates confidence interval; *IS*, ischemic stroke

**Table 3:** Multivariable conditional logistic regression model of risk factors for IS in non-diabetic and non-hypertensive young population

Parameters	B	SE	Wald	*P*	Exp (B)	95%CI
FT3, pmol/L	−2.945	.931	10.006	.002	.053	.008–.326
HDL, mmol/L	−7.134	2.367	9.083	.003	.001	.000–.083
HCY, μmol/L	.417	.135	9.574	.002	1.518	1.165–1.977
ANC, 10^9^/L	.883	.420	4.414	.036	2.418	1.061–5.511

*B* regression coefficient; *SE* standard error; Exp (B) indicates odds ratio (OR); *CI* indicates confidence interval; *IS* ischemic stroke. We used a forward stepwise logistic regression analysis with a statistical variable removal level of *P* < 0.10

## Discussion

Herein, we evaluated risk factors for early-onset IS among non-diabetic non-hypertensive patients. We found that IS patients exhibited elevated TG, HCY, WBC, ANC, SBP, and DBP levels, as well as lower HDL-C, TC, FT3, and FT4 levels relative to controls. Further analysis revealed that HCY and ANC levels were positively correlated with IS risk, whereas HDL-C and FT3 levels were negatively correlated with such risk.

Our findings are consistent with those of many other prior analyses which have detected negative correlations between HDL-C levels and IS incidence in young adults. Indeed, while low HDL-C has been linked to elevated IS risk in young populations, LDL-C, TG, and TC levels have been found to be unrelated to such risk [16–18]. These findings are not universal, however, as no significant relationship between HDL-C levels and IS was observed in the ARIC Study [19]. Indeed, some studies have found HDL-C levels to be negatively correlated with vascular events and mortality among IS patients [20, 21], and one study found elevated HDL-C levels to be correlated with decreased IS severity in patients ≤50 years old, whereas no such relationship was observed among older patients [22]. HDL-C is involved in reverse cholesterol transport, but also plays antithrombotic and anti-inflammatory roles [23, 24]. In one systematic review, the risk of IS was found to decrease by 11–15% for every 10 mg/dL increase in HDL-C [25]. HDL administration has also been found to be neuroprotective, as it is associated with reducing neutrophil recruitment and preserving the blood-brain barrier [26]. Based on our data and these prior studies, we can hypothesize that HDL-C play a key role in mediating IS risk among younger patients, and that preventative efforts aimed at elevating serum HDL-C levels in younger adults may significantly decrease IS risk.

We also found that FT3 levels were negatively correlated with IS incidence among young adults. This is the first study to our knowledge to have reported such a relationship, as prior studies and meta-analysis have largely focused on correlations between low FT3 levels and severity/mortality among acute IS patients [27–31]. These prior studies have reported such a relationship even for low FT3 values within the standard reference range [32, 33]. One study, however, found that this relationship was age-dependent, such that TT3 and FT3 levels were unrelated to IS outcomes in patients < 65 years old, whereas TT3 levels were independently predictive of poor outcomes among older patients [34]. In these prior studies, the average population ages ranged from 61.4–81.5 years [27–32], with the exception of one study with a median population age of 48 years [33]. Serum TT3 and FT3 levels are lower among healthy older individuals [35], and FT3 levels decline over time [36]. Age may, therefore, have the potential to confound the effect of FT3 on the cardiovascular system in older individuals, whereas lower FT3 levels in younger patients may be more clinically significant. Triiodothyronine (T3) has been shown neuroprotective effects with a possible mechanism of activation of nitric oxide signaling and vasodilation [37, 38]. Moreover, T3 modulates reorganization of neuronal circuits and synaptic plasticity, motor recovery and functional connectivity during critical periods of recovery after stroke [39]. T3 administration may reduce infarct volume and related edema through the inhibition of aquaporin-4 expression in perimicrovascular astrocytic endfeet [37, 40]. As acute diseases can decrease the conversion of FT4 to FT3 [41], reductions in FT3 levels in acute IS patients may simply be indicative of disease rather than a driver of disease progression. Future research will be essential in order to confirm the mechanistic basis for the observed correlation.

We additionally found that HCY levels were positively correlated with early-onset IS risk. Hyperhomocysteinemia is well known to be associated with elevated IS risk [42]. In line with our findings, many prior studies have confirmed elevated HCY levels to be associated with IS risk in younger patient populations [43–46], with this correlation being strongest in younger individuals [43]. The risk of IS has been shown to be roughly two-fold higher in IS patients with high HCY levels relative to patients with low HCY levels [47, 48]. Huang et al. in contrast, determined a 20% reduction in HCY level to be linked with a 7% decrease in IS risk [49]. HCY levels are also correlated with stroke severity, with a poorer prognosis [50, 51], and with stroke recurrence [52]. A 3 μmol/L decrease in total HCY levels was associated with a 10% reduction in the risk of stroke recurrence [53]. HCY may elevate the risk of IS incidence through mechanisms including the impairment of thrombolysis, thrombosis, enhanced H_2_O_2_ production, endothelial dysfunction, and increased LDL-C oxidation [54–56]. HCY, also, can favor atrial fibrillation occurrence and a subsequent IS (particularly in cardioembolic and cryptogenic strokes) by inducing an electrical and structural atrial remodelling [57, 58].

We also found ANC levels to be positively correlated with early-onset IS risk, in line with prior reports that ANC is an independent predictor of IS incidence and recurrence [59–61]. Other studies have also found elevated ANC values to be related to more severe stroke, poorer stroke outcomes, larger infarct volumes [62, 63], the presence of intracranial atherosclerotic stenosis [64], and unstable carotid plaques [65]. Neutrophils can influence thrombosis and atherosclerosis, thereby modulating the risk of IS [66, 67]. Neutrophil activation can be reduced by elevated levels of HDL-C and consequent changes in pro-inflammatory cytokine production [68], partially explaining the beneficial impact of HDL-C levels in this context. Neutrophils are key markers of acute inflammation, and are among the first cells recruited following IS [66]. Whether neutrophil accumulation is a response to stroke or a risk factor associated with IS incidence thus requires further investigation.

This study has multiple limitations compared with previous studies that younger strokes showed higher frequency of alcohol abuse, heavy smoking, obesity [69] and physical activity provided metabolic and anti-inflammatory properties [70]. For one, we were not able to account for all possible risk factors pertaining to IS incidence (including nutrition, alcohol intake, and physical inactivity) in this patient population owing to a lack of complete data. For another, we had not evaluated risk factors being influenced by IS subtype (e.g. TOAST classification [71]) distribution for lacking of complete subtypes data. It would be useful to assess the differences among subtypes of IS, particularly between cardioembolic and non-cardioembolic infarction because cardioembolic stroke is the subtype of IS with the highest in-hospital mortality and poor short-term prognosis [72]. Furthermore, we selected controls at random from among health patients, resulting in significant differences in rates of cigarette smoking between the case and control populations that may have significantly impacted the results of our analyses. This was also a retrospective single-center study with a small sample size, potentially biasing our results. Despite these limitations, we believe that these data represent a valuable first step towards understanding risk factors associated with early-onset IS incidence among non-hypertensive and non-diabetic patients.

## Conclusions

Rates of early-onset IS among non-hypertensive non-diabetic patients are rising globally. One of the future lines of research in the study of IS in young people would be its relationship with cerebrovascular risk factors. Our results suggest that HCY and ANC levels are positively correlated with the risk of early-onset IS, whereas HDL-C and FT3 levels were negatively correlated with such risk. These results provide a valuable framework for future research aimed at understanding how controlling these risk factors may prevent or reduce the risk of IS in younger populations.

## Data Availability

The data in this study are available from the corresponding author upon reasonable request.
